# Drug-induced sleep endoscopy-directed adenotonsillectomy in pediatric obstructive sleep apnea with small tonsils

**DOI:** 10.1371/journal.pone.0212317

**Published:** 2019-02-22

**Authors:** Jie Chen, Shan He

**Affiliations:** Department of Otolaryngology, Shanghai Children’s Medical Center Affiliated with Shanghai Jiaotong University School of Medicine, Shanghai, China; University of Connecticut Health Center, UNITED STATES

## Abstract

The study aims to examine drug-induced sleep endoscopy (DISE) in the decision-making process of pediatric obstructive sleep apnea (OSA) patients with small tonsils. This was a retrospective study of children who underwent awake flexible endoscopy, DISE, and adenoidectomy with/without tonsillectomy at the Shanghai Children’s Medical Center between 03/2015 and 12/2016. Tonsillectomy was performed for tonsillar obstruction found by DISE. Adenoidectomy was performed for all children. Cardio-pulmonary coupling (CPC) and oximetry were observed before/after surgery. The study included 126 children: 56 (44.4%) with grade 2 tonsils and 70 (55.6%) with grade 1. Mean age was 5.7±3.2 (range, 2.8–10.4) years and mean BMI of 15.7±5.5 kg/m^2^. Unexpectedly, DISE showed tonsillar obstruction in 57 (45.2%) children, including 44 (78.6%) with grade 2 tonsils and 13 (18.6%) with grade 1. Therefore, DISE-directed tonsillectomy was performed for 57 patients. There was an improvement in respiratory disturbance index (RDI) and oxygen saturation nadir in the DISE (P = 0.0007, P = 0.037) and control (P = 0.001, P = 0.023) groups 6 months after surgery, but RDI improvement was better in the DISE group compared with controls 1 year after surgery (P = 0.042). DISE is a good way to determine the necessity of tonsillectomy in pediatric OSA patients with small tonsils.

## Introduction

Obstructive sleep apnea (OSA) is a common childhood condition which can result in sequelae including growth retardation, poor school performance, enuresis, and behavioral problems [1]. Adenotonsillar hypertrophy is a major contributing factor to the development of OSA in children [2]. Adenotonsillectomy is the most commonly performed procedure for pediatric OSA, resulting in significant improvement or resolution of OSA severity. In our clinic, the gradation of tonsillar enlargement is assessed using the Brodsky tonsil grading scale [2]. According to the size of the tonsils, the tonsillar enlargement is classified into five levels. Tonsils of grades 3 and 4 are considered as tonsillar hypertrophy [3]. Tonsillectomy is commonly performed for children with tonsillar hypertrophy, but not for those whose tonsils are grade 1 or 2 without recurrent chronic tonsillitis. As the combined volume of the tonsils and adenoids is correlated with OSA severity [3], children with small tonsils may not experience the same benefit from tonsillectomy as the children whose tonsils are hypertrophic. Because of the different physiological status between sleep and wakefulness, oropharynx obstruction caused by small tonsils during sleep is uncertain.

Edwards et al. [4] found that the pharyngeal muscles reduce their activity during sleep. In children with an anatomically small airway, the pharyngeal muscles cannot always compensate for the increased mechanical load. Thus, a vulnerable situation in which the airway is prone to collapse may occur with the development of OSA. Whether small tonsils contribute to lateral pharyngeal wall obstruction during sleep is uncertain based on routine examinations. The challenge for the physician is identifying the cases of oropharynx obstruction caused by small tonsils in order to direct further interventions.

The diagnosis of OSA is confirmed by polysomnography (PSG), however, this evaluation does not provide any information regarding the specific anatomic sites of upper airway obstruction [5, 6]. Common assessments of the upper airway (such as physical evaluation or flexible fiberoptic laryngoscopy) are limited by the fact that these evaluations are typically static and performed during wakefulness, which may not accurately represent the dynamic upper airway collapse during sleep [7]. In order to develop the most targeted and effective surgical treatment plan, drug-induced sleep endoscopy (DISE) has been developed to characterize the location and pattern of upper airway obstruction [8, 9].

DISE is an evaluation technique that involves the assessment of individuals under pharmacologic sedation and is designed to simulate natural sleep [10]. DISE uses fiberoptic endoscopy to examine the upper airway [10]. It was first described by Croft and Pringle in 1991 [10]. Since then, many studies have shown DISE to be a safe and useful tool in the evaluation of upper airway obstruction [11–13].

During sleep endoscopy, dynamic airway obstruction can be observed at several levels [14, 15]. In particular, the aim of the present study was to examine the role of DISE in the decision-making process of pediatric OSA patients with small tonsils, in order to evaluate the necessity of tonsillectomy in pediatric OSA with small tonsils.

## Methods

### Study design and subjects

This was a retrospective study of children who underwent awake flexible endoscopy, DISE, and adenoidectomy with/without tonsillectomy at the Division of Otolaryngology of the Shanghai Children’s Medical Center, a tertiary pediatric hospital, between March 2015 and December 2016. This study was approved by the Institutional Review Board of the Shanghai Children’s Medical Center. The need for individual consent was waived by the committee.

The inclusion criteria were: 1) completed nocturnal oximetry monitor and cardio-pulmonary coupling (CPC) before and after both DISE and adenoidectomy with/without tonsillectomy; 2) preoperative respiratory disturbance index (RDI), collected from CPC, ≥5 events/hour in the presence of clinical signs or symptoms of OSA (frequent awakenings from sleep, loud snoring, pauses in breathing at night, and/or excessive daytime sleepiness); and 3) adenoid hypertrophy without tonsillar hypertrophy according to awake flexible endoscopy and oropharyngeal examination before surgery. The exclusion criteria were: 1) previous adenotonsillectomy; 2) any inherited syndrome; 3) craniofacial deformity; or 4) neurologic impairment. All patients were scheduled to undergo adenoidectomy according to the oropharyngeal examination, flexible endoscopy and CPC. Before general anesthesia, the oropharyngeal obstruction was assessed again by DISE. If oropharyngeal obstruction was caused by the tonsils, bilateral tonsillectomy would be performed at the same time as the adenoidectomy.

### Grouping

The patients were grouped according to the parents' choice: the DISE group and the control group. DISE represents additional medical procedure and expenses for the parents and they were left to decide whether or not they desired the procedure. The parents of all children in both groups had decided to perform adenoidectomy according to clinical routine examinations (oropharyngeal exam, fiber laryngoscopy, and CPC). Before surgery, we would inquire the parents whether they were willing a DISE examination during surgery, that was, before the induction of anesthesia. If they were, we would assign the child into the DISE group. For children who were found to be with oropharyngeal obstruction caused by the tonsils, we would recommend a simultaneous tonsillectomy. If the parents were unwilling to accept the DISE examination, we would assign the children into the control group.

### Evaluation of tonsil and adenoid size

The tonsil size was evaluated during a direct vision examination of the oropharynx. Tonsil size was evaluated preoperatively using the Brodsky grading scale [2]. Tonsils that did not extend beyond the tonsillar pillars were grade 1; tonsils that extended just beyond the tonsillar pillars were grade 2; tonsils that extended beyond the pillars but did not approach the midline were grade 3; and tonsils that touched or approached the midline were grade 4. Adenoid size was also quantified from 1 to 4 by flexible nasal endoscopy. When the entire vomer could be seen, the adenoids were graded as 1; when the bottom one-third of the vomer was obscured, they were graded as 2; when two-thirds of the vomer was obscured by adenoid tissue, they were graded as 3; and when the vomer was completely obscured by adenoidal tissue, they were graded as 4.

### Evaluation of tonsillar obstruction degree and surgical approach

The patients in the control group only underwent adenoidectomy. The surgical procedures for patients in the DISE group were determined according to the severity of oropharynx obstruction by the tonsil. The degree of tonsillar obstruction was determined through endoscopic/DISE according to a published tonsillar obstruction grading score [16]: no obstruction was grade A; 0–50% lateral obstruction was grade B; 50–99% lateral obstruction was grade C; and complete obstruction was grade D. In the present study, if the tonsil-induced oropharynx obstruction was grade A or grade B under DISE examination, then adenoidectomy was performed. If the tonsil was grade C or grade D, tonsillectomy and adenoidectomy were performed. We also referred to the tonsils with more than 2/3 of the volume located in the tonsillar fossa as endophytic tonsils.

### CPC

A heart rate recorder (Nanjing Fengsheng Yongkang Software Technology Co., Ltd., Nanjing, China) was placed on the patient’s chest to obtain the electrocardiogram (ECG) signal during sleep, using a single-lead ECG. An automated beat detection algorithm [17] was used to detect the beats and classify them as either normal or ectopic based on their morphology and timing [18, 19]. A CPC analysis derived RDI (CPC-RDI) was generated, using the duration and mean frequency of the low frequency coupling (LFC) periods, and expressed as number/h by multiplying two variables: 1) the average proportion of time (min/h) that a subject’s sleep was spent in the low frequency coupling state; and 2) the average frequency (cycles/min) of low frequency coupling [17, 18]. All children underwent CPC before surgery and at the 6^th^ month and 1^st^ year after surgery, at home. The changes of RDI before and after upper airway surgeries were analyzed.

### Nocturnal oximetry monitor

The nocturnal oximetry monitor (CloudCare Healthcare Co., Ltd.) has the appearance of a wrist watch and can be worn comfortably during sleep [20, 21]. The changes of oxygen saturation nadir collected from the oximetry monitor before and after upper airway surgery were compared.

### DISE

The patients in the DISE group underwent DISE in the supine position under general anesthesia in the operating room. No topical anesthesia was used before or during the procedure. A dexmedetomidine infusion was started with a loading dose of 2 μg/kg over 10 min, followed by a 2 μg/kg/h infusion. Ketamine was administered at 1–2 mg/kg at the start of the dexmedetomidine loading dose. Once a rhythmic pattern of respiration was established, a flexible fiberoptic laryngoscope was passed via the nares to the nasopharynx. The laryngoscope was then passed into the oropharynx, where the pharyngeal tonsils were examined to evaluate the dynamic airway obstruction.

### Statistical analysis

Statistical analysis was performed using SPSS 17.0 for Windows (IBM, Armonk, NY, USA). Continuous variables were expressed as mean ± SD. The Wilcoxon matched-pairs signed-rank test was used to compare RDI and oxygen saturation nadir before and after surgery. The results were considered statistically significant for P-values <0.05.

## Results

**Fig 1:**
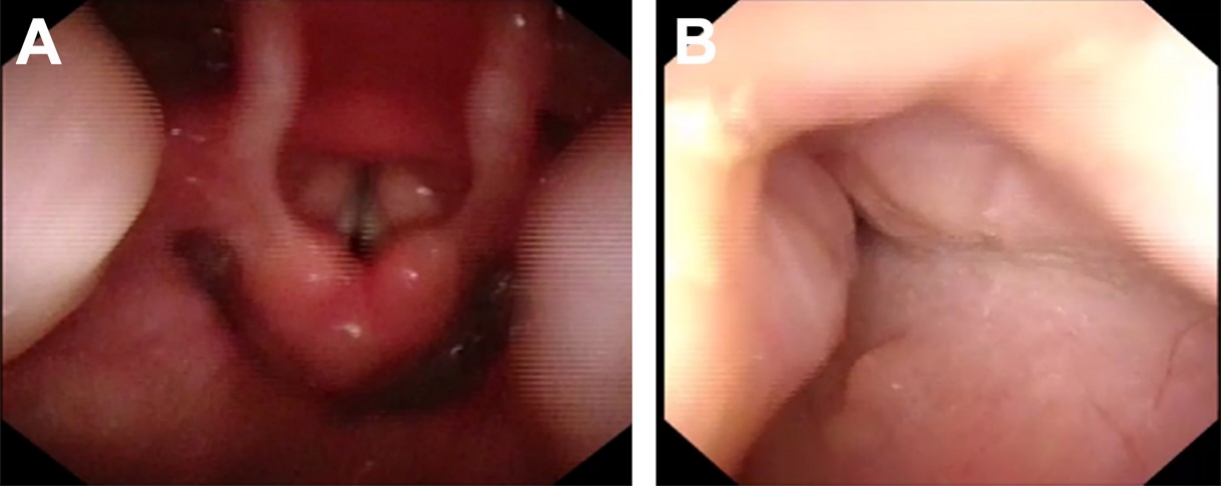
**(**A) A 6-year-old boy who underwent awake endoscopy. The oropharynx space did not collapse much and expanded during respiration. (B) The same child who underwent DISE. In the oropharynx, the airway was narrowed by the pharyngeal tonsils with medial dynamic displacement, leading to the almost complete obstruction of the airway on inspiration.

A total of 126 consecutive children who underwent awake endoscopy, DISE, and adenoidectomy with/without tonsillectomy were identified, and 200 patients were included in the control group (Table 1). In the DISE group, there were 42 females (33.3%) and 84 males (66.7%) with a mean age of 5.7±3.2 (range 2.8 to 10.4) years. The mean body mass index was 15.7±5.5 kg/m^2^. Oximetry monitor reports were reviewed for the oxygen saturation nadir. CPC reports were reviewed for the RDI. According to the awake endoscopy reports, the tonsillar obstruction of the 126 children was grade B. The tonsil size of 70 children (55.6%) was grade 1, and the tonsil size of 56 children (44.4%) was grade 2 (Table 1) according to Brodsky grading scale. There were no difference in adenoid size between the two groups (P = 0.35) (Table 1). According to the DISE reports, the tonsillar obstruction of 57 children turned into grade C. Among them, 44 (78.6%) was grade 2 tonsils and 13 (18.6%) was grade 1 tonsils. The tonsillar obstruction of 69 children was still grade B (Fig 1). In the DISE group, 7 (12.3%) children patients had endophytic tonsils among the grade 1 patients and 24 (42.1%) patients had endophytic tonsils among the grade 2 patients. Therefore, DISE-directed tonsillectomy was performed for 57 patients. Adenoidectomy was performed for grade B children, and adenotonsillectomy was performed for grade C children.

**Table 1:** Demographic data of the DISE and control groups.

	DISE group (n = 126)	Control group (n = 200)	P
Gender, Female, n (%)	42/126 (33.3%)	87/200 (43.5%)	0.068
Age, years, mean (^a^SD)	5.7 (3.2)	7.3 (3.7)	0.812
Body mass index, kg/m^2^, mean (SD)	15.7 (5.5)	17.2 (4.4)	0.523
Tonsil size, grade 1/grade 2, n (%), oropharyngeal examination	70/56 (55.6/44.4)	92/108 (46.0/54.0)	0.093
Adenoid, grades C/D	67/59	117/83	0.35
^b^RDI events/hour, preoperative (SD)	13.7 (8.5)	11.3 (5.1)	0.52
RDI events/hour, 6 month after surgery(SD)	2.9 (1.4)	3.5 (2.3)	0.47
RDI events/hour, 1 year after surgery(SD)	4.2 (3.7)	5.7 (3.4)	0.04
oxygen saturation nadir %, preoperative(SD)	84.5 (11.5)	83.2 (9.6)	0.33
oxygen saturation nadir %, 6 month after surgery(SD)	94.3 (6.3)	95.8 (3.9)	0.36
oxygen saturation nadir %, 1 year after surgery (SD)	92.5 (6.6)	89.1 (7.1)	0.25

^a^SD = standard deviation

^b^RDI = respiratory disturbance index.

The results of tonsil size identified with awake endoscopy are different from that by DISE. We observed a change in 45.2% of the initial treatment proposal.

**Fig 2:**
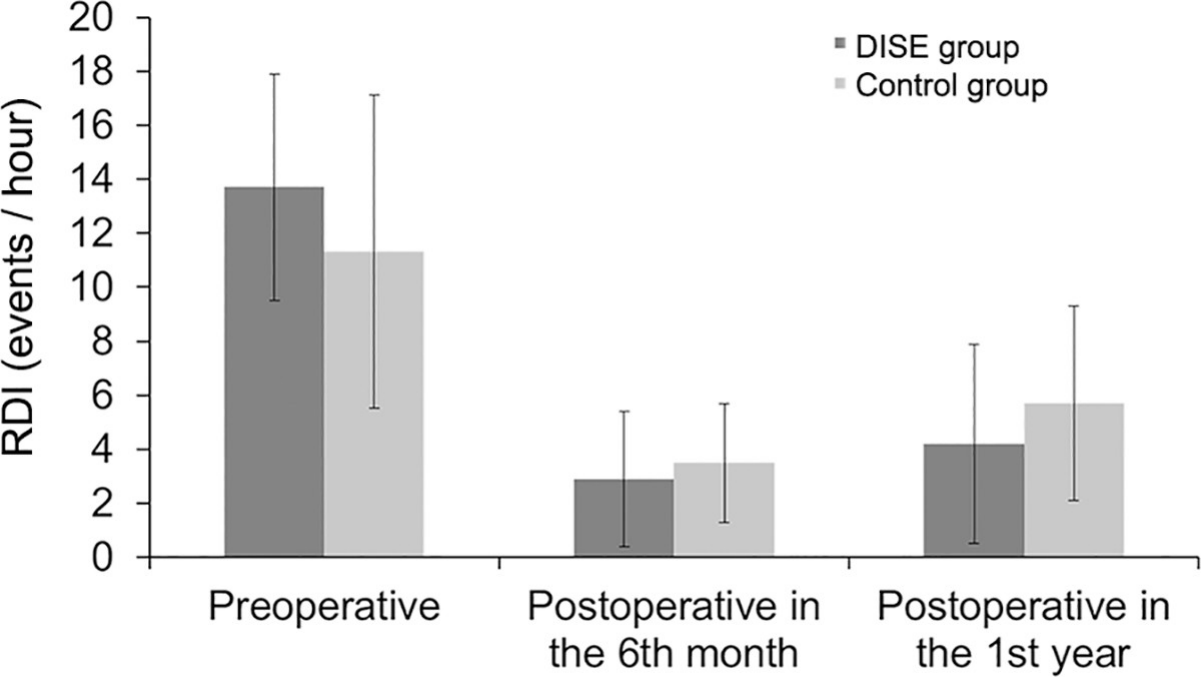
Respiratory disturbance index (RDI) before and after surgery in children with OSA who underwent adenoidectomy with/without tonsillectomy, according to DISE influencing or not the surgical management (P<0.05).

**Fig 3:**
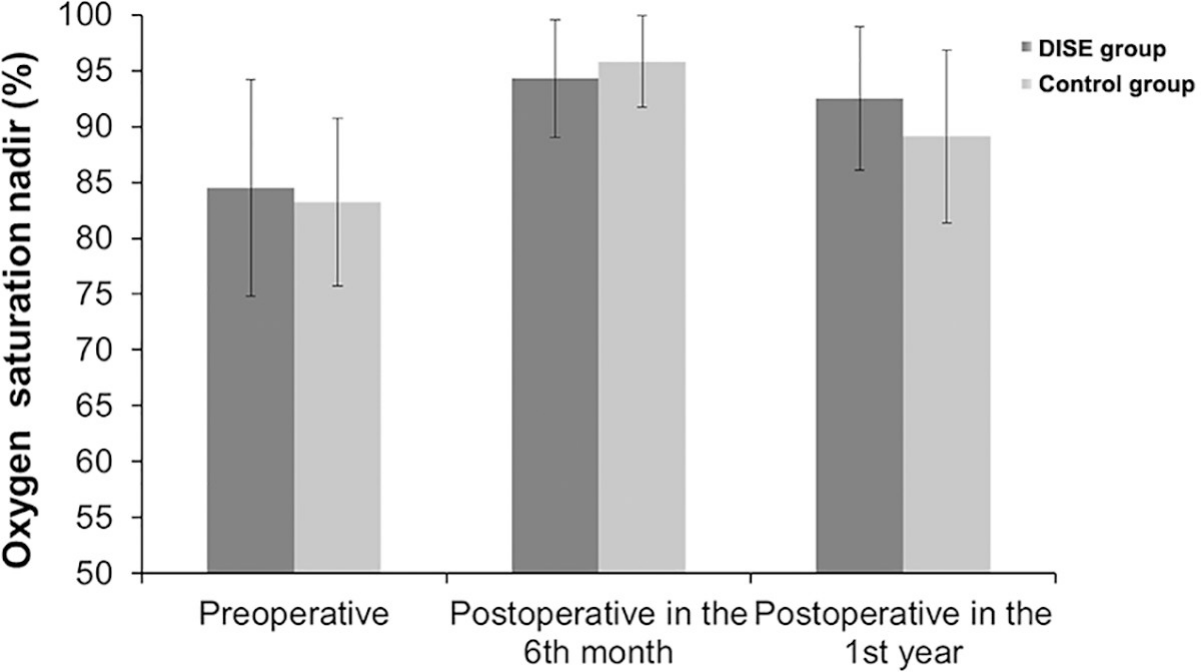
Oxygen saturation nadir index before and after surgery in children with OSA who underwent adenoidectomy with/without tonsillectomy, according to DISE influencing or not the surgical management.

Post-operative CPC and nocturnal oximetry monitor were performed in the 6^th^ month and the 1^st^ year after surgery. RDI and oxygen saturation nadir were compared between the DISE and control groups. Postoperative RDI of the DISE and control groups in the 6^th^ month (2.9±1.4 events/hour, 3.5±2.3 events/hour) and 1^st^ year (4.2±3.7 events/hour, 5.7±3.4 events/hour) were significantly improved compared with preoperative RDI (13.7±8.5 events/hour, 11.3±5.1 events/hour), although some obstructions may still be present (Fig 2). The RDI was better improved in the DISE group compared with control group in the 1^st^ year after surgery (P = 0.042). Oxygen saturation nadir was improved in both groups in the 6^th^ month (94.3±6.3%, 95.8±3.9%) and the 1^st^ year (92.5±6.6%, 89.1±7.1%) after surgery compared with preoperative values (84.5±11.5%, 83.2±9.6%) (P = 0.037, P = 0.023) (Fig 3).

There were two children who had secondary postoperative hemorrhage. There was no other postoperative complication in the two groups.

## Discussion

Adenotonsillectomy is the first-line treatment for children with OSA, but it is uncertain whether children with small tonsils experience the same benefit as those with tonsil hypertrophy. Therefore, the aim of the present study was to examine the role of DISE in the decision-making process of pediatric OSA patients with small tonsils. Because we were not sure whether there are differences in curative effects between adenoidectomy alone and DISE-mediated tonsillectomy and adenoidectomy, we assigned the children who did not receive DISE into the control group, and only adenoidectomy was performed after the oropharyngeal examination, fiberoptic laryngoscopy, and CPC evaluation. The follow-up results showed that the RDI was better improved in the DISE group compared with the control group in the 1^st^ year after surgery. In addition, the results showed that the unexpected DISE findings were that regions of tonsillar obstruction occurred in 57 (45.2%) children. In the DISE group, we found that the tonsillar obstruction of 57 children turned into grade C. Therefore, we performed adenotonsillectomy instead of adenoidectomyin these children only. Although 45.2% children in the DISE group had a different surgery, the RDI was better improved in the DISE group compared with the control group at 1 year after surgery (P = 0.042, significantly different between the DISE and control groups). Therefore, DISE is a good way to determine the necessity of tonsillectomy in pediatric patients with OSA and small tonsils.

In most Chinese hospitals, if a child is diagnosed with OSA and has a clinical examination consistent with adenotonsillar hypertrophy, the clinician recommends adenotonsillectomy as the first line of treatment. If the child has OSA with adenoid hypertrophy but without tonsil hypertrophy or recurrent pharyngeal tonsil infection, adenoidectomy alone is commonly recommended. It is generally considered that tonsils that are grade 1 or 2 in size may not contribute to airway obstruction in children [3]. The traditional routine examination (such as ENT examination, X-ray cephalometry, and awake endoscopy) has been found to be incomplete and unable to detect the obstruction during sleep. When awake, the increased muscle tone may offer a false appearance regarding airway obstruction [22], as high pharyngeal neuromuscular tone can prevent the collapse of upper airways during wakefulness. Sleep onset results in a progressive upper airways muscular hypotonia due to the reduction of the neurophysiologic phenomenon, which is more common in patients with OSA than in normal people [23]. This process contributes to a partial or even a complete obstruction in the oropharynx of patients with OSA. The awake state is quite different from the sleep situation, so inaccurate information may lead to inappropriate treatment. In order to determine the necessity of tonsillectomy for OSA patients with small tonsils, DISE is warranted. It provides a dynamic assessment of the oropharynx to determine the optimal treatment of OSA while mimicking natural sleep.

As there is no uniform protocol for DISE sedation, different kinds of anesthetic agents are used. A majority of studies reported that inhalational anesthetics and opioids exaggerate the dynamic airway collapse, suggesting that they are not the best choice for DISE [24]. Sleep induced using propofol and remifentanil is not a perfect model for natural sleep either, because they can cause excessive hypotonia and muscle relaxation with altered airway dynamics resulting in an inaccurate model of natural slumber [25]. A recent systematic review suggested that dexmedetomidine and ketamine do not lead to respiratory depression; they are associated with less muscular relaxation, with a more sustained respiratory effort [26]. At our center, the combination of dexmedetomidine and ketamine are routinely used because it is considered that they provide a better simulacrum of sleep and consequently more accurate diagnosis. Nevertheless, different drug regimens could be tested in a future study.

As sleep centers with pediatric expertise are limited, waiting times are often prolonged, the waiting list rapidly grows, and the need for multiple sleep studies makes OSA diagnosis and management in children particularly challenging. At our pediatric sleep center, PSG is the gold standard test for children with OSA, however, there are other screening tests when PSG is not available. Other screening tests include nocturnal oximetry monitoring to observe the change of blood oxygen saturation during sleep, and CPC to obtain the RDI. CPC is based on single-lead ECG recording during sleep. It first extracts normal sinus rhythm time series, and the respiration time series are derived from the ECG; then the degree of coherence and the cross spectrum of these two signals are calculated using the Hilbert-Huang transformation technology to finally obtain the sleep clinical stage [17, 18]. CPC can precisely identify the clinical stages of sleep, and the results were verified to be consistent with traditional electroencephalogram-based sleep study [18].

In children with tonsillar hypertrophy, there is lateral crowding of the oropharynx exacerbated by medialization of the lateral pharyngeal walls during inspiration. In some children with small tonsils, especially endophytic ones, the lateral pharyngeal medialization results in similar central crowding. In the present study, the degree of oropharynx obstruction was significantly increased when observed with DISE compared to awake endoscopy. The most relevant difference between DISE and awake endoscopy lies in the underestimation of the degree of the collapse during wakefulness. There was no big difference detected in obstruction configurations. Based on the above findings, adenoidectomy may not be sufficient for OSA children with small tonsils, because small tonsils may contribute to persistent obstruction during sleep, although they seem not to cause any obstruction in the awake state.

Our study has several limitations. Since the growing demand and the need for multiple sleep studies makes OSA diagnosis and management particularly challenging, parameters from PSG, which is the gold standard to diagnose OSA, was not consistently used in the children included in the present study and could not be included in the analyses. Nevertheless, the validity of CPC and nocturnal oximetry monitoring for OSA diagnosis has been reported [17, 18, 20, 21]. In addition, the study was done at a single tertiary medical center. In the future, data collection from multiple centers would provide data that could be more easily generalized.

The information acquired from routine ENT examinations during wakefulness may be inaccurate and underestimate the degree of obstruction of the oropharynx. This can lead to incorrect evaluation of obstruction caused by small tonsils. Preoperative assessment combining DISE could have better outcomes than routine ENT examinations in pediatric OSA patients with small tonsils. Although there was no difference in DISE-mediated tonsillectomy at postoperative 6 months compared to the control group, our study has found that the improvement of OSAS clinical symptoms by DISE-mediated tonsillectomy is higher compared to the control group at 1 year of follow-up, which may be associated with the obstruction of the upper airway by the tonsils during longer follow-up.

## Supporting information

S1 Dataset Initial data. (XLSX)
